# From Rapid Progression to Long-Lasting Complete Response: A Wide Range of Ripretinib Activity for Metastatic GIST—A Real-World Cohort

**DOI:** 10.3390/medsci14050549

**Published:** 2026-09-07

**Authors:** Hanna T. Frumin Edri, Walid Shalata, Ilia Berezhnov, Tanzila Tairov, Tatiana Bobrovitsky, Dan Mirelman, Esther Tahover, Ofer Purim, Yuval Dadon, Katerina Shulman, Gali Perl, Gil Bar Sela, Maria Passhak, Ronen Brenner

**Affiliations:** 1 Oncology Institute, Edith Wolfson Medical Center, Holon 58220, Israel; 2 Faculty of Medicine, Tel Aviv University, Tel Aviv 69978, Israel; 3 Legacy Heritage Cancer Center, Soroka Medical Center, Beer Sheva 84105, Israel; 4 Medical School for International Health, Faculty of Health Sciences, Ben Gurion University of the Negev, Beer Sheva 84105, Israel; 5 Tel Aviv Sourasky Medical Center, Tel Aviv 69978, Israel; 6 Department of Oncology, Assuta Medical Center, Ramat HaHayal, Tel Aviv 63293, Israel; 7 Faculty of Life Sciences, Bar Ilan University, Ramat Gan 69978, Israel; 8 Helmsley Cancer Center, Shaare Zedek Medical Center, Jerusalem 91031, Israel; 9 Hebrew University of Jerusalem, Jerusalem 91031, Israel; 10 Carmel Medical Center, Haifa 34362, Israel; 11 Davidoff Cancer Center, Rabin Medical Center, Petah Tikva 49414, Israel; 12 Technion Integrated Cancer Center, Faculty of Medicine, Technion, Haifa 31096, Israel; 13 Department of Oncology, Emek Medical Center, Afula 18341, Israel; 14 Rambam Health Care Campus, Haifa 35254, Israel

**Keywords:** GIST, ripretinib, real-world evidence, KIT mutation, switch-control kinase inhibitor

## Abstract

**Background**: Ripretinib, a switch-control kinase inhibitor approved for advanced gastrointestinal stromal tumor (GIST), inhibits primary and secondary *KIT* and *PDGFRA* mutations. Real-world data from Western and Middle Eastern populations remain limited. We report outcomes from a molecularly characterized Israeli multicenter cohort. **Methods**: We conducted a multicenter retrospective cohort study across seven university-affiliated medical centers in Israel. Patients with advanced GIST receiving Ripretinib at any treatment line were eligible. Progression-free survival (PFS) and overall survival (OS) were estimated using the Kaplan–Meier method, and response was assessed according to modified RECIST v1.1. **Results**: Sixteen patients were identified (median age, 67.5 years; 75.0% received Ripretinib in the fourth-line setting). KIT exon 11 mutations were present in 62.5% of patients, including five with secondary resistance mutations involving exon 13 or exons 17/18. Median PFS was 7.0 months (95% CI, 2.0–13.0) and median OS was 14.7 months (95% CI, 4.4–NR). Individual outcomes varied substantially, with PFS ranging from 2.0 to 60.2 months and OS from 2.2 to 60.2 months. Three patients achieved complete responses, with PFS of 20.0, 33.6, and 60.2 months. ORR was 56.3% (CR, *n* = 3; PR, n = 6) and DCR was 81.3%. No dose reductions or treatment discontinuations due to adverse events were documented; however, adverse events were recorded in only 4 of 16 patients. **Conclusions**: Ripretinib demonstrated clinical activity in this small, heavily pre-treated cohort. The relatively high ORR and wide variability in outcomes should be interpreted cautiously given the small sample size, retrospective design, and absence of central radiological review. Durable responses were observed in some patients with KIT exon 11 and secondary exon 17/18 mutations, but this exploratory observation does not establish a predictive molecular subgroup. Larger prospective studies with systematic molecular, radiological, and safety assessment are warranted.

## 1. Introduction

Gastrointestinal stromal tumors (GISTs) are the most common mesenchymal malignancies of the gastrointestinal tract, with a global incidence of approximately 10–15 cases per million per year [1,2]. The majority harbor activating mutations in the *KIT* proto-oncogene (~75% of cases) or *PDGFRA* gene (~10%), with *KIT* exon 11 representing the most prevalent subtype [3,4]. Sequential TKI therapy has transformed the prognosis of advanced GIST: imatinib (first-line), sunitinib (second-line), and regorafenib (third-line) each provide incremental benefit, with median PFS of approximately 18–24, 5–6, and 4–5 months, respectively [5,6,7,8,9].

Resistance to each TKI is driven by heterogeneous secondary *KIT* mutations in the ATP-binding domain (exons 13/14) or activation loop (exons 17/18) [6,7,8,9]. Sunitinib is more active against exon 13/14 than exon 17/18 variants; regorafenib has limited activity against both. This mutational heterogeneity has historically left patients beyond the third-line with few effective options. Ripretinib (Qinlock^®^, Deciphera Pharmaceuticals) addresses this through a switch-control mechanism: by simultaneously occupying the switch pocket and activation loop, it locks *KIT* and *PDGFRA* in an inactive conformation, enabling broad inhibition of primary and secondary mutations regardless of exon location [10,11,12,13,14,15,16].

In the pivotal phase III INVICTUS trial (n = 85 Ripretinib; ≥4th-line GIST), Ripretinib achieved a median PFS of 6.3 months versus 1.0 month with placebo (HR 0.15; *p* < 0.0001) and a median OS of 15.1 versus 6.6 months (HR 0.36) [12]. An ORR of 9.4% and DCR of 23.5% were reported. The phase III INTRIGUE trial subsequently demonstrated comparable PFS to sunitinib in the second-line setting (8.0 vs. 8.3 months) with superior tolerability and similar OS (35.5 vs. 31.5 months) in the final analysis [13,17,18,19,20,21], and ctDNA biomarker analyses identified compound *KIT* exon 11 + activation-loop (exon 17/18) mutations as a subgroup deriving substantially greater benefit from Ripretinib versus sunitinib (ORR 44.4% vs. 0%; PFS 14.2 vs. 1.5 months) [13,14].

Because GIST is rare and patients reach fourth-line therapy only after sequential attrition through imatinib, sunitinib, and regorafenib, the population eligible for Ripretinib in routine practice is exceedingly small. Robust real-world data in this heavily pre-treated, molecularly selected setting are correspondingly scarce—and, outside East Asia, almost absent.

Published real-world cohorts are largely confined to a Chinese prospective registry (n = 240; mPFS 7.7 months) [16], a smaller Chinese observational study (n = 23; mPFS 7.1 months) [17], a Taiwan/Hong Kong compassionate-use cohort (n = 20; mPFS 6.1 months) [20], and a UK expanded access program (EAP) cohort (n = 45; mPFS 7.9 months) [18], the only published non-Asian real-world experience to date. We report here an Israeli multicenter retrospective cohort study examining the efficacy and tolerability of Ripretinib in routine clinical practice, with outcomes compared directly with landmark clinical trials and published real-world series.

## 2. Materials and Methods

### 2.1. Study Design and Patient Population

This is a multicenter, retrospective, observational cohort study conducted across seven university-affiliated medical centers in Israel: Edith Wolfson Medical Center (Holon), Rambam Health Care Campus (Haifa), Emek Medical Center (Afula), Davidoff Cancer Center, Rabin Medical Center (Petah Tikva), Assuta Medical Center (Ramat HaHayal), Tel Aviv Sourasky Medical Center, and Lin Medical Center (Haifa). All adult patients (≥18 years) with histologically confirmed advanced GIST who received at least one dose of Ripretinib 150 mg once daily (QD) were eligible, regardless of treatment line, performance status, or mutational profile. For assessment of treatment response and disease control, patients were required to have at least one post-baseline clinical assessment. Data were retrieved by retrospective chart review with a data cutoff of 1 March 2026.

### 2.2. Endpoints and Definitions

Progression-free survival (PFS): Time from first Ripretinib dose to radiological or clinical disease progression, or death from any cause. Patients without events were censored at their last clinical assessment. Overall survival (OS): Time from first Ripretinib dose to death from any cause; survivors were censored on 1 March 2026. ORR: Proportion achieving CR or PR per mRECIST v1.1. DCR: Proportion achieving CR, PR, or stable disease (SD). Adverse events were graded per CTCAE v5.0.

### 2.3. Statistical Analysis

Continuous variables are reported as median and range, and categorical variables as frequency and percentage. Survival was estimated by the Kaplan–Meier method. Subgroup analyses by KIT mutational status (exon 11 vs. other/unknown) are presented descriptively. Given the small sample size, formal hypothesis testing was not performed. Analyses were conducted using Python 3.12 with the lifelines library (v0.27). Ethics committee approval was obtained at each participating center; the study was conducted in accordance with the Declaration of Helsinki.

## 3. Results

### 3.1. Patient Characteristics

Sixteen patients with advanced GIST received Ripretinib between March 2021 and the data cutoff of 1 March 2026, across the seven participating medical centers. Baseline characteristics are summarized in Table 1; individual patient outcomes in Table 2. The median age at Ripretinib initiation was 67.5 years (range 29.7–86.9), with 62.5% aged ≥ 65 years. Nine patients (56.3%) were female. Primary tumor sites included the small bowel/jejunum (43.8%), stomach (25%), duodenum (6.3%), and other or unknown sites (25%). Six patients (37.5%) had metastatic disease at initial diagnosis; the remainder developed metastatic progression after initial resection.

KIT/PDGFRA molecular profiling was available in 12/16 patients (75%). KIT exon 11 was the primary mutation in 10 patients (62.5%), of whom five had secondary mutations detected at progression on prior lines of therapy, presumed acquired resistance mutations involving the exon 13 (n = 3) or exon 17/18 activation loop (n = 3, including one patient with concurrent exon 17 and exon 18 mutations). Patient 1 harbored primary KIT exon 11 without identified secondary mutations. Two patients (12.5%) had primary KIT exon 9 mutations; molecular data were unavailable in four patients (25.0%). Histologic grade was high in seven patients (43.8%), low in one (6.3%), and unknown in eight (50.0%). Ripretinib was administered as a fourth-line in 12 patients (75.0%), a third-line in two patients (12.5%), and a sixth-line in two (12.5%). All had previously received imatinib; most had also received sunitinib and/or regorafenib. Eight patients (50.0%) were alive at the data cutoff.

### 3.2. Efficacy Outcomes

**Figure 1:**
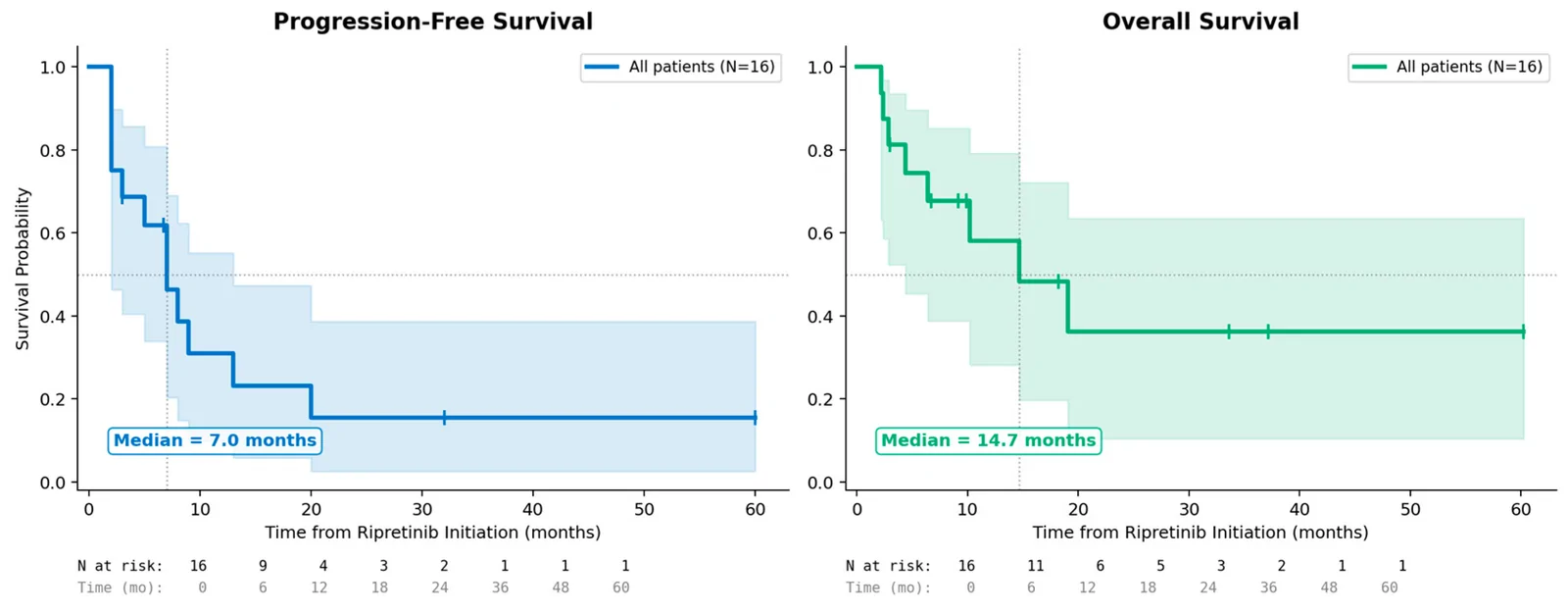
Kaplan–Meier curves for progression-free survival and overall survival (N = 16). Progression-free survival (PFS; **left panel**, median 7.0 months, 95% CI 2.0–13.0) and overall survival (OS; **right panel**, median 14.7 months, 95% CI 4.4-NR) from Ripretinib initiation. Shaded areas represent 95% confidence intervals. Tick marks (|) indicate censored observations (patients alive or progression-free at data cutoff, 1 March 2026). Numbers at risk shown below each panel.

At the data cutoff, four patients (25.0%) remained on Ripretinib and were censored for the PFS analysis. Efficacy outcomes are summarized in Table 3, cross-study comparisons in Table 4, and survival curves are shown in Figure 1.

#### 3.2.1. Progression-Free Survival

**Figure 2:**
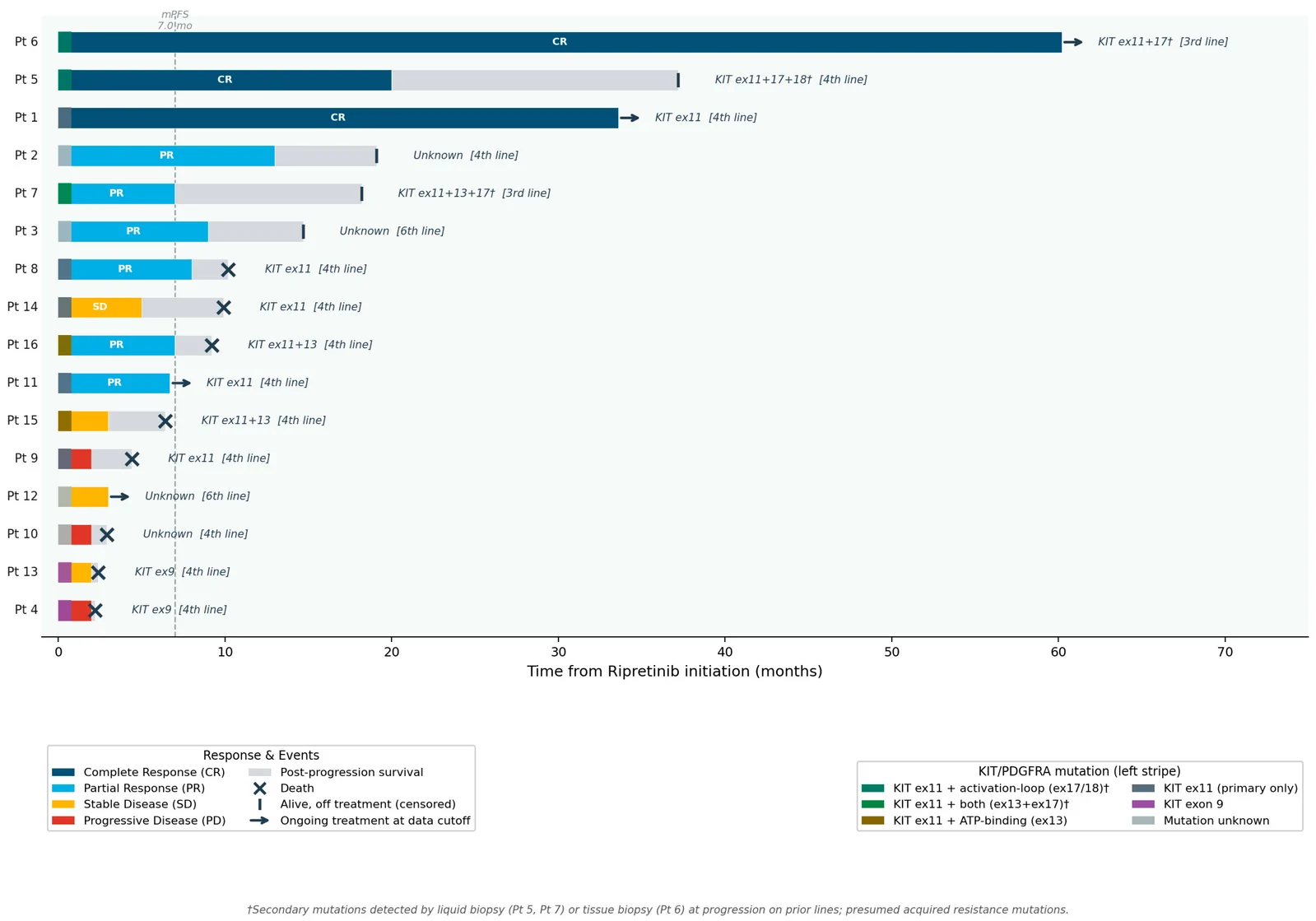
Ripretinib treatment duration and survival by best overall response (N = 16). Swimmer plot showing Ripretinib treatment duration and post-progression survival for each patient, sorted by overall survival (longest to shortest). Colored bars: CR = complete response (dark teal); PR = partial response (light blue); SD = stable disease (amber/gold); PD = progressive disease (red). Gray bars = post-progression survival. Left-edge stripes = KIT/PDGFRA mutational subgroup.

Median PFS was 7.0 months (95% CI 2.0–13.0; range 2.0–60.0. Three patients achieved PFS ≥ 20 months. The longest PFS, 60 months, was in patient 6, receiving Ripretinib as third-line therapy for KIT exon 11 + exon 17 mutations, who remained in ongoing complete response at the data cutoff. Four patients had PFS < 3 months; two harbored KIT exon 9 mutations, one with KIT exon 11 and one had no available molecular data (Figure 2).

#### 3.2.2. Overall Survival

Median OS from Ripretinib initiation was 14.7 months (95% CI 4.4-NR; range 2.2–60.2. Eight patients (50.0%) died during follow-up. Eight patients remained alive at the data cutoff, including four who were still receiving Ripretinib.

Tumor response: Among 16 evaluable patients, best overall responses were CR in three patients (18.8%), PR in six patients (37.5%), SD in four (25.0%), and PD as best response in three (18.8%). The ORR (CR + PR) was 56.3% (9/16) and the DCR (CR + PR + SD) was 81.3% (13/16). Among KIT exon 11 patients, the ORR was 70.0% (7/10). All three complete responses occurred in patients with KIT exon 11-mutated or compound exon 11 + activation-loop mutations, and all three were durable (20, 32, and 60 months), (Table 3).

### 3.3. Safety

Adverse event data were documented in 4 of 16 patients. The low rate of reported events is consistent with the retrospective design, where only clinically notable toxicities requiring action were systematically recorded. Fatigue/asthenia was the most frequent event (two patients; one grade 3). Other events included bone and muscle pain, nausea, loss of appetite, rash, facial paresthesia with scalp pruritus, and epigastric pain (each grade 1–2), and hypercalcemia of an undetermined grade in one patient. No dose reductions or treatment discontinuations due to adverse events were documented in this cohort, and no treatment-related deaths occurred (Table 5).

## 4. Discussion

This multicenter retrospective cohort of 16 patients with advanced, pre-treated GIST demonstrates a median PFS of 7.0 months and a median OS of 14.7 months with Ripretinib. No dose reductions or treatment discontinuations due to toxicity were documented, although safety findings should be interpreted cautiously given the limited adverse-event documentation. Placed against the landscape of published Ripretinib data (Table 4), these results are broadly consistent with pivotal trial findings and published real-world evidence.

**Efficacy outcomes**: The median PFS of 7.0 months and OS of 14.7 months are consistent with INVICTUS [12] and the 6.1–7.9 months PFS range across published real-world cohorts [16,17,18,20], supporting the consistency of these findings across different populations. The aggregate medians, however, mask substantial variability in individual outcomes, with PFS ranging from 2.0 to 60.2 months and OS from 2.2 to 60.2 months. CR patients achieved PFS of 20.0, 33.6, and 60.2 months (OS 37.2, 33.6, and 60.2 months), while PR patients had PFS of 6.7–13.0 months, SD of 2.0–5.0 months, and PD of 2.0 months. Ripretinib therefore produced a broad range of individual outcomes, from durable disease control, including responses lasting several years in some patients, to limited clinical activity. The observed association between the depth and durability of response is of interest but should be considered exploratory and requires confirmation in larger cohorts.

**Objective response rate**: The ORR of 56.3% was higher than that reported in INVICTUS (9.4%) [12], the Chinese real-world cohort (9.5%) [17], the UK EAP (16.7%) [18], and INTRIGUE (21.8%) in the second-line setting [13]. The DCR of 81.3% was also higher than that reported in INVICTUS (23.5%) [12]. These findings should, however, be interpreted cautiously given the small sample size, potential selection bias inherent to a retrospective cohort, and the absence of central radiological review. Several factors may have contributed to the observed response rate. Our cohort was enriched for patients with primary *KIT* exon 11 mutations (62.5% of the overall cohort and 83% of profiled patients), broadly comparable to INVICTUS and the UK EAP. Among patients with *KIT* exon 11 mutations, acquired activation-loop mutations in exon 17/18 and ATP-binding cleft mutations in exon 13/14 were each present in 30% of patients. Previous analyses have suggested that the activity of Ripretinib may vary according to the secondary *KIT* mutation profile, with particularly favorable outcomes reported in patients with *KIT* exon 11 plus exon 17/18 mutations [14,19,22]. Nevertheless, the small number of patients in each molecular subgroup prevents firm conclusions regarding genotype-specific treatment benefit. Therefore, the high ORR observed in our cohort should be regarded as hypothesis-generating and warrants confirmation in larger, independently assessed cohorts.

Two patients in our cohort—Pt 5 (*KIT* exon 11+17+18) and Pt 6 (*KIT* exon 11+17)—had the molecular profile associated with favorable Ripretinib activity in the INTRIGUE analysis, and both achieved complete responses, with PFS of 20.0 months (OS 37.2 months, fourth-line) and 60.2 months (OS 60.2 months, third-line), respectively. These observations are notable but should be interpreted in the context of the very small number of patients and do not establish a predictive molecular subgroup. By contrast, Pt 7, harboring primary *KIT* exon 11 combined with mutations in exons 13, 17, and 18 simultaneously, achieved only a partial response (PFS 7 months). This complex genotype does not map neatly to either INTRIGUE dichotomy (exon 11+17/18 or exon 11+13/14), although no conclusions regarding the effect of co-occurring resistance mutations can be drawn from a single patient. Notably, Pt 1 achieved 33.6 months of complete response with primary *KIT* exon 11 and no identified secondary mutations, possibly reflecting occult mutations not detected by available biopsy. In addition, 12.5% of patients received Ripretinib in the third-line setting, where response rates may be intrinsically higher, and molecular profiling was unavailable in 25% of patients, introducing ascertainment bias and limiting mutational landscape characterization. Moreover, the possibility that pharmacokinetic or pharmacodynamic differences may contribute to variations in treatment outcomes between this Middle Eastern cohort and predominantly East Asian or UK cohorts remains hypothetical and was not evaluated in the present study.

These real-world observations provide supportive, hypothesis-generating evidence relevant to the ongoing phase III INSIGHT trial (NCT05734105), which is prospectively evaluating Ripretinib versus sunitinib in imatinib-treated patients with *KIT* exon 11 + 17/18 mutations identified by ctDNA [23]. While our findings cannot establish the clinical relevance or predictive value of this molecular subgroup, the observed durable responses in some patients provide a rationale for further prospective evaluation of this genotype–treatment association. International collaborative efforts to pool molecular and clinical outcome data across diverse populations may further clarify *KIT* mutation frequencies, resistance patterns, and the potential role of molecular profiling in treatment selection for advanced GIST.

**Safety**: The documented toxicity profile—including fatigue, musculoskeletal symptoms, nausea, and skin changes—was qualitatively consistent with the established safety profile of Ripretinib reported in INVICTUS and INTRIGUE [12,13]. Although no dose reductions or treatment discontinuations due to adverse events were documented in our cohort, this finding should not be interpreted as evidence of favorable tolerability, as adverse events were recorded in only 4 of 16 patients. The low rate of documented AEs likely reflects the limitations of retrospective toxicity reporting and may underestimate the true incidence and severity of treatment-related toxicity. A pharmacovigilance analysis of the FAERS database identified additional safety signals, including cutaneous squamous cell carcinoma, liver abscess, and prostatomegaly, which were not observed in our small cohort but warrant prospective monitoring [22]. Given the small sample size and retrospective design, our safety findings should therefore be considered descriptive and interpreted cautiously.

Although numerically small, this cohort should be interpreted in the context of disease rarity and the line of therapy. With a GIST incidence of only ~10–15 per million per year and progressive attrition across prior lines, the fourth-line advanced GIST population is small; sixteen patients accrued across seven national centers therefore provide real-world experience from this clinically narrow population. The detailed molecular and individual outcome data may provide useful hypotheses for further investigation, although the small sample size limits statistical power, generalizability, and the ability to draw definitive genotype-outcome conclusions.

**Limitations**: As expected for a rare disease treated in the fourth-line setting, the sample size was small (n = 16), precluding multivariable analyses and definitive conclusions regarding potential biomarkers. Nevertheless, the cohort provides detailed molecular characterization and individual genotype-outcome data that may be useful for hypothesis generation. The retrospective design introduces potential selection bias, incomplete adverse-event documentation, and variability in the availability and quality of clinical data. In particular, the histological grade was unavailable in approximately 50% of patients, while molecular profiling was unavailable in 25% of patients, which may have limited our ability to fully characterize disease risk and account for potential prognostic factors. The low rate of documented adverse events in this cohort contrasts with the broader spectrum of safety signals reported in large pharmacovigilance datasets [22], underscoring the importance of prospective safety monitoring. Finally, treatment response was not centrally reviewed according to mRECIST v1.1 or by a blinded independent review committee, which may have introduced assessment variability.

## 5. Conclusions

Ripretinib demonstrated clinical activity in this small cohort of patients with advanced, heavily pre-treated GIST, with a median PFS of 7.0 months and OS of 14.7 months, broadly consistent with findings from INVICTUS and published real-world cohorts. Patients with primary KIT exon 11 mutations and acquired exon 17/18 activation-loop resistance mutations included several patients with particularly durable responses, including complete responses lasting several years. Although this observation is consistent with previous reports suggesting activity of Ripretinib in this molecular context, the small number of patients precludes definitive conclusions regarding a predictive molecular subgroup. Safety findings should also be interpreted cautiously, as adverse events were documented in only 4 of 16 patients and the retrospective design may have resulted in under-reporting of toxicity. No unexpected safety signals were identified, but the limited adverse-event documentation does not allow firm conclusions regarding the tolerability of Ripretinib in this cohort. Systematic molecular re-profiling at progression, using tissue or ctDNA when appropriate, may provide clinically relevant information regarding the evolving resistance landscape. Larger prospective studies and multicenter registries with systematic molecular and safety characterization are needed to determine whether specific secondary KIT mutation profiles are associated with differential benefit from Ripretinib and to better define its optimal treatment positioning in advanced GIST.

## Figures and Tables

**Table 1:** Baseline patient and disease characteristics (N = 16).

Characteristic	N	%
**Age at Ripretinib initiation (years)**		
Median (range)	67.5 (29.7–86.9)	-
<65 years	6	37.5
≥65 years	10	62.5
**Sex**		
Male	7	43.8
Female	9	56.3
**Primary tumor site**		
Small bowel/jejunum	7	43.8
Stomach	4	25.0
Duodenum	1	6.3
Other/unknown	4	25.0
**Stage at initial diagnosis**		
Non-metastatic	10	62.5
Metastatic at diagnosis	6	37.5
**Histologic grade**		
High grade	7	43.8
Low grade	1	6.3
Unknown	8	50.0
**Primary *KIT*/*PDGFRA* mutation**		
*KIT* exon 11	10	62.5
*KIT* exon 9	2	12.5
Unknown/not done	4	25.0
**Secondary *KIT* mutations † (among *KIT* exon 11 patients, n = 10)**		
*KIT* exon 13 (V654A)	3	30.0
*KIT* exon 17 (Y823D/N822K)	3	30.0
*KIT* exon 18 (A829P)	1	10.0
**Ripretinib treatment line**		
3rd line	2	12.5
4th line	12	75.0
6th line	2	12.5
**Status at data cutoff (1 March 2026)**		
Alive	8	50.0
Deceased	8	50.0

Notes: † Secondary mutations (exon 13, 17, or 18) were detected by liquid biopsy (patients 5 and 7) or tissue biopsy (patient 6) at progression on prior lines; presumed acquired TKI resistance mutations rather than co-primary alterations. Five of the 10 KIT exon 11 patients harbored secondary mutations: exon 13 (n = 3; Pts 7, 15, 16), exon 17 (n = 3; Pts 5, 6, 7), and exon 18 (n = 1; Pt 5). Patients 5 and 7 each carry mutations in two secondary exons simultaneously, hence per-exon counts exceed unique patient counts.

**Table 2:** Individual patient outcomes.

Variable	Pt 1	Pt 2	Pt 3	Pt 4	Pt 5	Pt 6	Pt 7	Pt 8	Pt 9	Pt 10	Pt 11	Pt 12	Pt 13	Pt 14	Pt 15	Pt 16
**Primary tumor site**	Stomach	Stomach	Duodenum	Small bowel	Small bowel	Jejunum	Stomach	Unknown	Small bowel	Jejunum	Small bowel	Abdomen	Small bowel	Stomach	Unknown	Small bowel
**Molecular profile**	*KIT* ex11	Unknown	Unknown	*KIT* ex9	*KIT* ex11 + 17 + 18 †	*KIT* ex11 + 17 †	*KIT* ex11 + 13 + 17 †	*KIT* ex11	*KIT* ex11	Unknown	*KIT* ex11	Unknown	*KIT* ex9	*KIT* ex11	*KIT* ex11 + 13	*KIT* ex11 + 13
**Line of therapy**	4th	4th	6th	4th	4th	3rd	3rd	4th	4th	4th	4th	6th	4th	4th	4th	4th
**BOR**	CR	PR	PR	PD	CR	CR	PR	PR	PD	PD	PR	SD	SD	SD	SD	PR
**PFS (months)**	33.6	13	9	2	20	60.2	7	8	2	2	6.7	3	2	5	3	7
**OS (months)**	33.6	19.1	14.7	2.2	37.2	60.2	18.2	10.2	4.4	2.9	6.7	3	2.4	9.9	6.4	9.2

Abbreviations: Pt = patient number; BOR = best overall response; CR = complete response; PR = partial response; SD = stable disease; PD = progressive disease; PFS = progression-free survival; OS = overall survival. † Secondary mutations (exon 13, 17, or 18) detected by liquid biopsy (Pts 5 and 7) or tissue biopsy (Pt 6) at progression on prior lines; presumed acquired resistance mutations.

**Table 3:** Summary of efficacy outcomes (N = 16).

Outcome	Result
**Progression-Free Survival (N = 16)**	
Median PFS, months	7.0
95% CI, months	2.0–13.0
Range, months	2.0–60.0
Patients censored (ongoing at data cutoff)	4 (25.0%)
**Overall Survival (N = 16)**	
Median OS from Ripretinib initiation, months	14.7
95% CI, months	4.4-NR
Range, months	2.2–60.2
Deaths at data cutoff	8 (50.0%)
**Tumor Response (N = 16 evaluable)**	
Complete response (CR)	3 (18.8%)
Partial response (PR)	6 (37.5%)
Stable disease (SD)	4 (25.0%)
Progressive disease (PD)	3 (18.8%)
Objective response rate (ORR; CR + PR)	56.3% (9/16)
Disease control rate (DCR; CR + PR + SD)	81.3% (13/16)
ORR in *KIT* exon 11 subgroup (n = 10 evaluable)	70.0% (7/10)

Abbreviations: n, number of patients; CI, confidence interval; CR, complete response; DCR, disease control rate; KIT, KIT proto-oncogene, receptor tyrosine kinase; NR, not reached; ORR, objective response rate; OS, overall survival; PD, progressive disease; PFS, progression-free survival; PR, partial response; SD, stable disease. INTRIGUE mOS (35.5 months, Ripretinib arm) is from the final OS analysis (data cutoff March 2023); PFS and ORR are from the primary analysis.

**Table 4:** Cross-study comparison of Ripretinib efficacy outcomes.

Study (Year)	N	Setting	mPFS (mo)	mOS (mo)	ORR (%)	DCR (%)
INVICTUS ph.III RCT (Blay et al., 2020) [12]	85	≥4th line	6.3	15.1	9.4	23.5
Phase I study (Janku et al., 2020) [15]	83	≥4th line	5.6	NR	11.3	NR
INTRIGUE ph.III RCT (Bauer et al., 2022; Heinrich et al., 2025) [13,21]	163	2nd line	8.0	35.5	21.8	68.7
Chinese registry (Zhang X et al., 2023) [16]	240	≥4th line	7.7	NR	NR	NR
Chinese RW cohort (Yang et al., 2023) [17]	23	≥3rd line	7.1	12.0	9.5	85.7
UK EAP (Lim et al., 2024) [18]	45	≥3rd line	7.9	14.0	16.7	66.7
Taiwan/HK CU (Lin et al., 2022) [20]	20	≥4th line	6.1	NR	25.0	60.0
This cohort (2026)	16	3rd–6th line	7.0	14.7	56.3	81.3

**Table 5:** Treatment-related adverse events.

Adverse Event	Any Grade n (%)	Grade 1–2 n (%)	Grade ≥ 3 n (%)
Fatigue/asthenia	2 (12.5)	1 (6.3)	1 (6.3)
Bone and muscle pain	1 (6.3)	1 (6.3)	0
Nausea	1 (6.3)	1 (6.3)	0
Loss of appetite	1 (6.3)	1 (6.3)	0
Rash	1 (6.3)	1 (6.3)	0
Facial paresthesia/scalp pruritus	1 (6.3)	1 (6.3)	0
Epigastric pain	1 (6.3)	1 (6.3)	0
Hypercalcemia	1 (6.3)	-	-
Any adverse event (≥1 AE documented)	4 (25.0)	-	1 (6.3)
Dose reduction due to AE	0	-	-
Treatment discontinuation due to AE	0	-	-

Abbreviations: n, number of patients; AE, adverse events. Note: Adverse events graded per CTCAE v5.0. Low documentation rate reflects retrospective design; only clinically notable events were systematically recorded.

## Data Availability

The original contributions presented in this study are included in the article. Further inquiries can be directed to the corresponding author.

## Abbreviations

The following abbreviations are used in this manuscript:

Abbreviation	Definition
AE	Adverse event
BOR	Best overall response
CI	Confidence interval
CR	Complete response
CTCAE	Common Terminology Criteria for Adverse Events
DCR	Disease control rate
EAP	Expanded access program
FAERS	FDA Adverse Event Reporting System
GIST	Gastrointestinal stromal tumor
HR	Hazard ratio
KIT	KIT proto-oncogene, receptor tyrosine kinase
mOS	Median overall survival
mPFS	Median progression-free survival
mRECIST	Modified Response Evaluation Criteria in Solid Tumors
NR	Not reached
ORR	Objective response rate
OS	Overall survival
PD	Progressive disease
PDGFRA	Platelet-derived growth factor receptor alpha
PFS	Progression-free survival
PR	Partial response
QD	Once daily
RCT	Randomized controlled trial
RECIST	Response Evaluation Criteria in Solid Tumors
